# Evaluation of measles outbreak response in Geze Gofa district, South Ethiopia region, using the 7–1–7 timeliness metrics

**DOI:** 10.1186/s12879-026-13299-2

**Published:** 2026-04-16

**Authors:** Lensa Fekadu, Abduilahafiz Hassen, Habtamu Yimer, Donatus Nbonibe Abaane, Tinsae Eshetu, Jafer Kezali, Assefa Tola

**Affiliations:** 1 Ethiopian Public Health Institute, Addis Ababa, Ethiopia; 2 Resolve To Save Lives, Ethiopia Office, Addis Ababa, Ethiopia; 3 Ministry of Health, Addis Ababa, Ethiopia; 4 Africa Center for Disease Control and Prevention, Addis Ababa, Ethiopia; 5 School of Public Health, College of Health and Medical Sciences, Haramaya University, Harar, Ethiopia; 6 Public Health Emergency Management (PHEM) Expert at EPHI – PHEM, Intermediate Field Epidemiology Training Program Resident, Ethiopia Filed Epidemiology and Laboratory Training Program (EFELTP), Addis Ababa, Ethiopia

**Keywords:** Measles, Outbreak response, 7–1–7 framework, Timeliness, Ethiopia, Public health surveillance, Vaccine coverage

## Abstract

**Background:**

Measles remains a highly contagious, vaccine-preventable disease with significant morbidity, particularly among under-immunized populations. Despite routine immunization and Supplementary Immunization Activities (SIAs), Ethiopia continues to experience recurrent outbreaks. The 7-1-7 framework provides benchmarks for timely outbreak detection, notification, and early response actions. This study aimed to describe measles outbreak epidemiology and evaluate response timeliness using 7-1-7 framework in Geze Gofa District of Southern Ethiopia.

**Methods:**

A mixed-methods field investigation was conducted from January to February 2025. Data were collected through active case finding, health facility record reviews, interviews with healthcare workers and caregivers, and observation of vaccination documentation. The 7–1–7 framework was applied to assess the timeliness of detection, notification, and response. Descriptive statistics were used to analyze case demographics and response timelines, while thematic analysis was employed to identify bottlenecks and enablers.

**Results:**

A total of 39 measles cases were identified, predominantly children aged 1–14 years (82.1%). Overall district attack rate was 0.37 per 1000 population [95% CI: 0.27–0.51], highest among children 0–4 years (1.11 per 1000 population [95% CI: 0.65–1.78]. The outbreak was notified within 1 day, meeting the 7–1–7 benchmarks. However, detection took place 11 days after the first case emergence and response activities were initiated in full at 11 days, both of which exceeded the target of 7 days. The delay in detection was mainly due to the absence of active surveillance in the community, and the response was further delayed by the need for laboratory confirmation, which took 10 days. Additional challenges included rugged terrain, limited vaccine supply, and insufficiently trained personnel. Despite these barriers, early notification were facilitated by trained health workers at outpatient service department and effective mobile communication systems. Notably, 13% of cases were unvaccinated, and an additional 12.8% had unknown vaccination status, despite reported MCV1 and MCV2 coverage rates of 96% and 94%, respectively in Geze Gofa District.

**Conclusion:**

The Geze Gofa measles outbreak response demonstrated strengths in notification but was hindered by delayed detection and response initiation due to systemic and logistical constraints. To enhance outbreak preparedness and align with the 7–1–7 framework, active surveillance should be strengthened, and national protocols should be revised to allow provisional vaccination based on clinical and epidemiological evidence. Decentralizing laboratory services and strengthening vaccine logistics are also critical to improving response timeliness and effectiveness.

**Supplementary Information:**

The online version contains supplementary material available at 10.1186/s12879-026-13299-2.

## Introduction

Measles is a highly contagious vaccine-preventable disease caused by the measles virus, an enveloped, single-stranded, negative-sense RNA virus of the genus *Morbillivirus* (family *Paramyxoviridae*). Effective control requires very high population immunity, usually achieved with about 95% coverage with two doses of measles-containing vaccine (MCV1 and MCV2) to interrupt transmission and sustain herd protection [1]. With an estimated basic reproduction number (R₀) of 12–18, a single infectious case can transmit the virus to many susceptible individuals [2]. Transmission occurs via respiratory droplets, and more than 90% of susceptible persons develop systemic infection characterized by fever, malaise, cough, coryza, and conjunctivitis, followed by a generalized maculopapular rash [3].

Measles is associated with frequent and severe complications. Pneumonia develops in up to 1 in 20 cases and is the leading cause of measles-related death, while acute encephalitis occurs in about 1 in 1,000 cases and blindness in roughly 1 in 1,000 patients, particularly among children with vitamin A deficiency. A rare but almost invariably fatal late complication, subacute sclerosing panencephalitis (SSPE), may occur years after infection in approximately 1 in 10,000 cases. These complications are especially common in children under five years of age, with up to 30% of reported cases in this age group experiencing at least one serious complication [4–6].

Measles continues to pose a serious public health threat worldwide, more than 50 years after vaccine introduction. The World Health Organization (WHO) reported outbreaks in every region in 2024–2025, including previously measles-free areas, underscoring persistent gaps in timely detection and response [7–9]. Although measles is vaccine-preventable, it remains a leading cause of vaccine-preventable childhood mortality, particularly in low-resource settings [8].

The burden is disproportionately high in sub-Saharan Africa, especially in conflict-affected, under-immunized, and resource-constrained settings [8]. By mid-2025, WHO surveillance documented over 188,000 suspected measles cases in the African Region [6]. Ethiopia has been significantly affected, experiencing repeated large-scale outbreaks and measles-associated deaths driven by low routine immunization coverage, high population mobility, and limited public health response capacity [10–12]. In Ethiopia, the region formerly known as the Southern Nations, Nationalities, and Peoples’ Region (SNNPR) has been reorganized into four regions, including the South Ethiopia Region. This region has significantly contributed to the national measles case count; a recent systematic review and meta-analysis found that approximately 13.4% (nearly 30,000) of all confirmed measles cases in Ethiopia from 2000 to 2023 originated from this area [13]. Despite extensive preventive and control efforts including nationwide and subnational vaccination campaigns, routine immunization through the Expanded Program on Immunization (EPI), supplementary immunization activities (SIAs) targeting high-risk populations, strengthened case-based surveillance, case management and public awareness campaigns measles transmission persists in Ethiopia [14]. The South Ethiopia Region, which includes the two study kebeles in this evaluation, has experienced recurrent measles outbreaks in recent years, making it a critical setting for assessing outbreak detection and response performance.

To strengthen epidemic preparedness and response, Ethiopia has adopted the “7–1–7” framework, a global standard designed to improve the timeliness of responses to infectious disease outbreaks [15–17]. The 7–1–7 target sets clear performance benchmarks: detecting a suspected outbreak within seven [8] days of its emergence, notifying public health authorities and initiating an investigation within one [1] day of detection, and initiation of effective response measures within seven [8] days of notification. This framework, developed by Resolve to Save Lives and endorsed by the WHO Regional Office for Africa as part of the Regional Strategy for Health Security and Emergencies 2022–2030, aims to enhance accountability, save lives, and accelerate readiness for future pandemics [15–17]. An effective outbreak response under this framework encompasses seven core components: (1) rapid deployment of a response team (2), epidemiological investigation (3), laboratory confirmation (4), case management and infection prevention and control (IPC) (5), implementation of public health countermeasures in affected communities (6), risk communication and community engagement, and (7) activation of coordination mechanisms to align partners and resources [18].

Although Ethiopia has formally adopted the 7–1–7 framework to guide rapid outbreak detection and response, there is limited evidence on how well these targets are being achieved at the district level during active outbreaks [19]. This study aims to fill that gap by evaluating the timeliness of detection, notification, and response efforts using the 7-1-7 framework for a recent measles outbreak in Geze Gofa district in January and February 2025, using the 7-1-7 framework.

## Methods

### Study setting and period

**Fig. 1:**
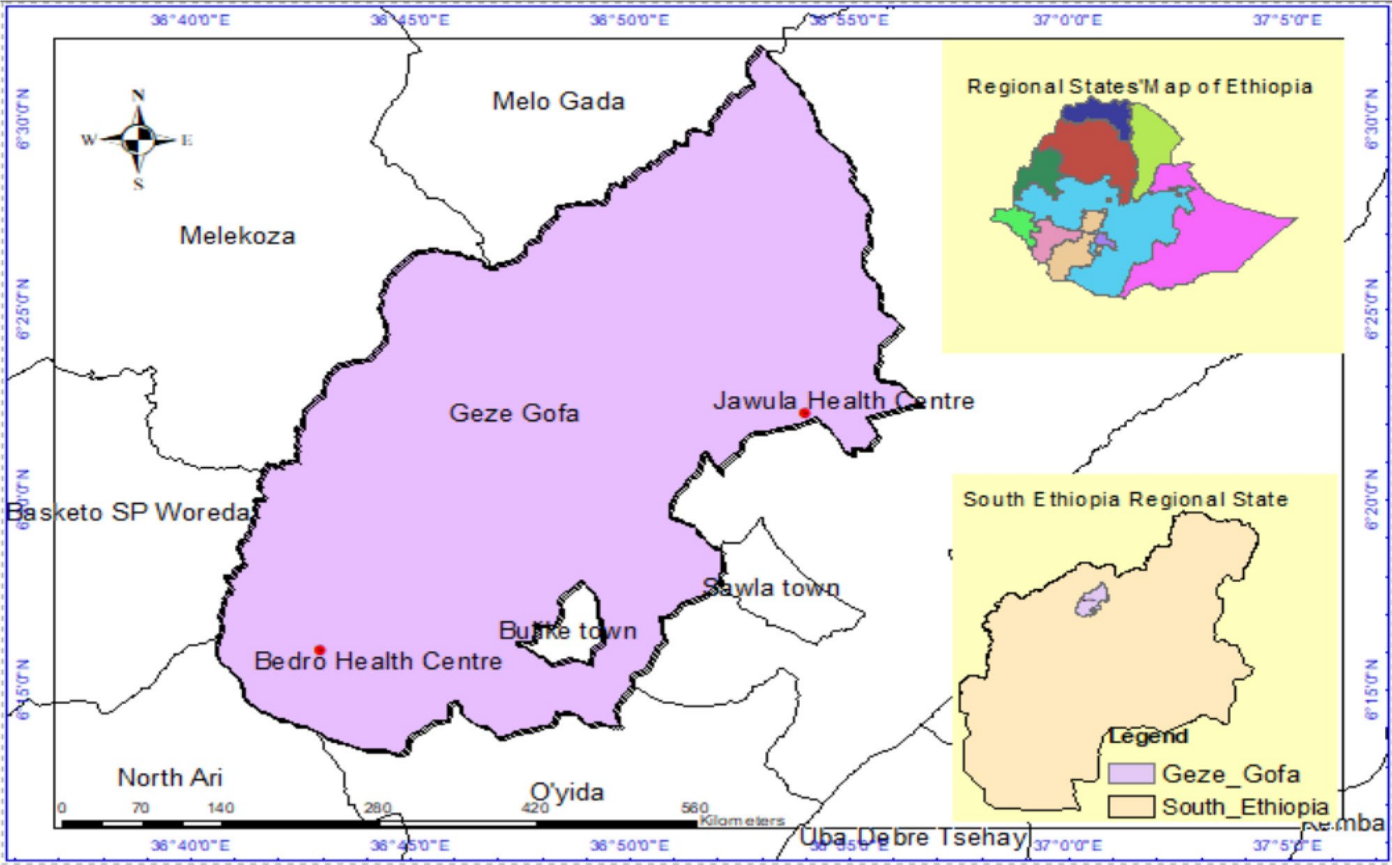
Map of Geze Gofa district

The measles outbreak was reported in two kebeles of Geze Gofa district: Jawula Uga and Bedro Germa. Geze Gofa is one of 97 districts in the South Ethiopia Regional State in Ethiopia, and has an estimated population of 104,575 people, including 15,310 children under five years of age [20]. The district faces a history of measles outbreak in April 2010. The district has no hospital, four health centers and 27 health posts (Fig. 1). The investigation was conducted from January to February 2025.

### Study design

A mixed-methods approach was employed to evaluate the measles outbreak response in the Geze Gofa District, integrating both quantitative and qualitative methodologies.

### Case definitions

A suspected case was defined as any person with fever and rash with one of the following: Cough, runny nose, and red eyes in Geze Gofa District from January 4, 2025, to February 24, 2025.

A confirmed case was defined as a suspected case that has been confirmed by laboratory testing of serum (positive IgM antibody) in Geze Gofa District from January 4, 2025, to February 24, 2025.

### Data collection process

On 16 January 2025, the Geze Gofa District Health Office notified the zonal PHEM unit of a suspected measles outbreak. triggering deployment of a five-member multidisciplinary investigation team (two Africa Epidemic Service fellows, two EPHI staff, one Intermediate Field Epidemiology resident).

The team conducted an active case search in Jawula Uga and Bedro Germa kebeles. House-to-house visits and reviews of outpatient and inpatient registers at two health centers identified additional cases. A standard measles case definition was applied. For each case, age, sex, address, date of rash onset, vaccination status, and outcome were abstracted. Vaccination cards were checked and caregivers interviewed about symptom onset and care-seeking. In total, 39 suspected and confirmed cases were identified, including three found only through active search. Seventeen key informant interviews were conducted using unstructured guides: health workers [4], patients [2], caregivers [8], the district PHEM officer [1], health center directors [2], and immunization focal persons [2], purposively selected to capture clinical,

The district preparedness was assessed to respond to public health emergencies using the 7-1-7 metrics. The response timeliness was evaluated starting from the initial detection to the early initiation of outbreak response activities. This start-to-finish evaluation assessed how quickly the district detected the outbreak, notified and implemented response measures. To gain insights into the response process, the 7–1–7 framework was used to document enabling factors and bottlenecks [18].

### Data analysis

A descriptive analysis of case characteristics was conducted. Quantitatively, we modeled the frequency of reported cases using Poisson methods appropriate for count data in outbreak settings. Qualitative data were then systematically reviewed to ensure accuracy, credibility and faithful representation of participant perspectives. Two members of the investigation team independently read transcripts and field notes multiple times and identified emerging themes through manual coding, with particular attention to experiences of outbreak detection and response. Illustrative quotations were selected to highlight key findings and enhance the authenticity of the results. Finally, quantitative and qualitative findings were integrated to provide a comprehensive understanding of the outbreak and the timeliness of the response.

## Results

Geze Gofa district is one of the six districts in southern Ethiopia region where the measles outbreak occurred during our deployment. A total of 39 measles cases were found in two kebeles of the Geze Gofa districts in south Ethiopia region. Thirty (76.9%) of the cases were found in Jawula Uga kebele and the remaining nine cases were found in Bedro Germa kebele.

The outbreak occurred from epidemiologic week 1 to week 7 of 2025, featuring sustained transmission following the onset of the primary case on January 4, 2025. A total of 39 cases were reported, with 21 (53.8%) being female and 18 (46.2%) males. Notably, no measles-related deaths were recorded during this period.

**Fig. 2:**
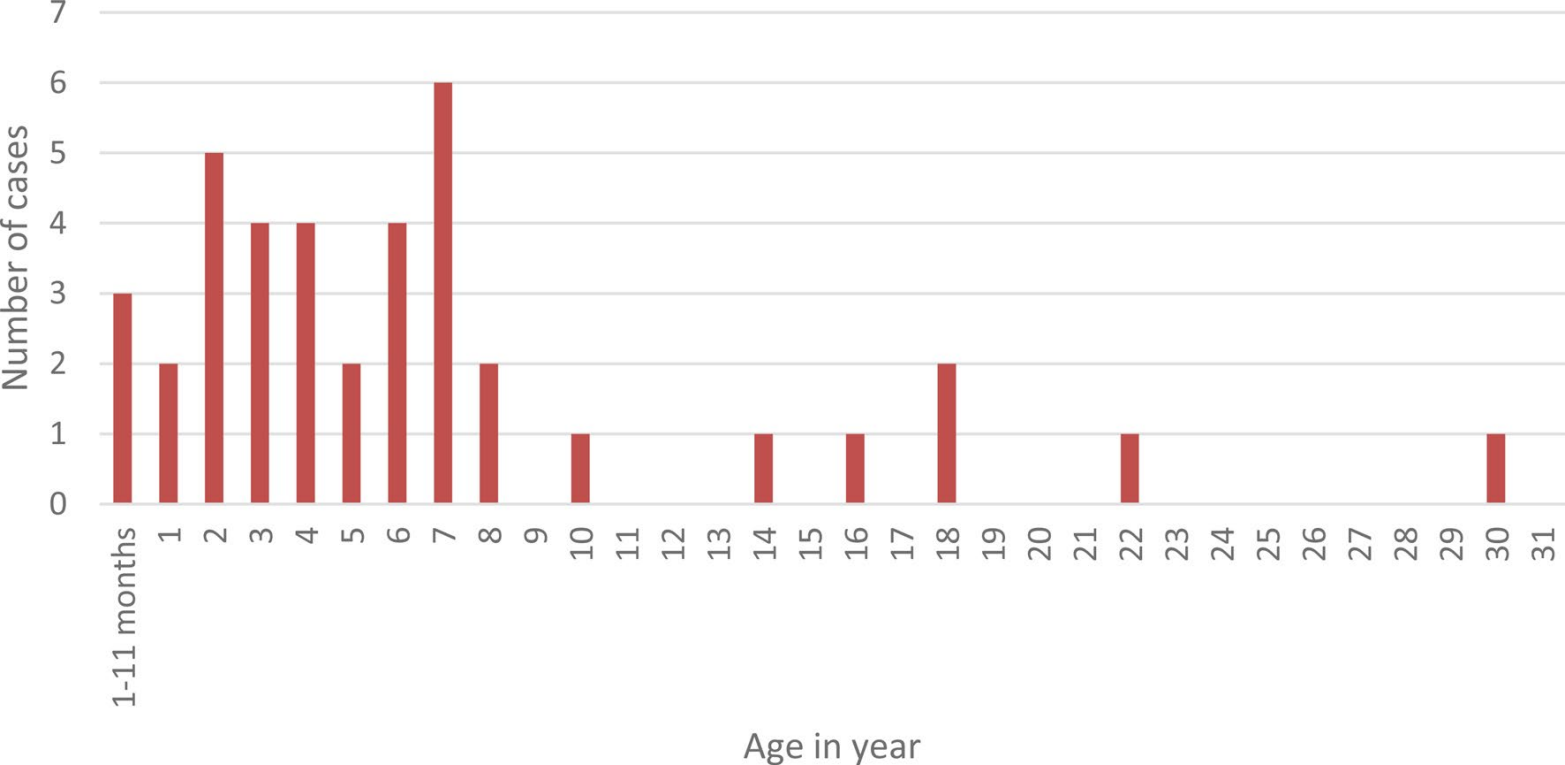
Age distribution of measles cases in Geze Gofa district, South Ethiopia region, February 2025

The ages of measle cases ranged from 3 months to 30 years, with a mean (± SD) of 6.94 (± 5.27) years (Fig. 2). Majority of cases occurred among children aged 1–14 years, accounting for 32 cases (82.1%) of all reported cases, of which 17 (43.6%) were between 5 and 14 years old.

Age distribution attack rate varies. Among children aged 0–4 years, there were 17 reported cases out of a population of 15,310, resulting in an attack rate of 1.11 per 1,000 population, which is significantly higher than the rates observed in older age groups. The 5–14-year age group also reported 17 cases, but with a larger population of 31,759, yielding a lower attack rate of 0.54 per 1,000. In individuals aged 15 years and older, the attack rate dropped sharply to 0.09 per 1,000, with only 5 cases identified in a population of 57,506. Furthermore, sex-specific attack rates indicated that males under five years of age experienced a higher rate (1.33 per 1,000) compared to females in the same age group (0.90 per 1,000)(Table 1).

**Table 1:** Measles attack rates by age and sex, Geze Gofa district, South Ethiopia, 2025

Age Group (Years)	Sex	Cases	Population (2025 projection)	Attack Rate per 1000 Population	95% Confidence Interval (per 1000 population)
0–4	Male	10	7,550	1.33	(0.75–2.18)
	Female	7	7760	0.90	(0.47–1.56)
	**Total**	**17**	**15**,**310**	**1.11**	**(0.65–1.78)**
5–14	Male	7	15,661	0.45	(0.21–0.87)
	Female	10	16,098	0.62	(0.34–1.07)
	**Total**	**17**	**31**,**759**	**0.54**	**(0.31–0.86)**
≥ 15	Male	1	28,356	0.04	(0.01–0.20)
	Female	4	29,150	0.14	(0.05–0.30)
	**Total**	**5**	**57**,**506**	**0.09**	**(0.03–0.20)**
Total		39	104,575	0.37	(0.27–0.51)

*Poisson exact 95% confidence intervals calculated on case counts and scaled to rates per 1000 population

**Fig. 3:**
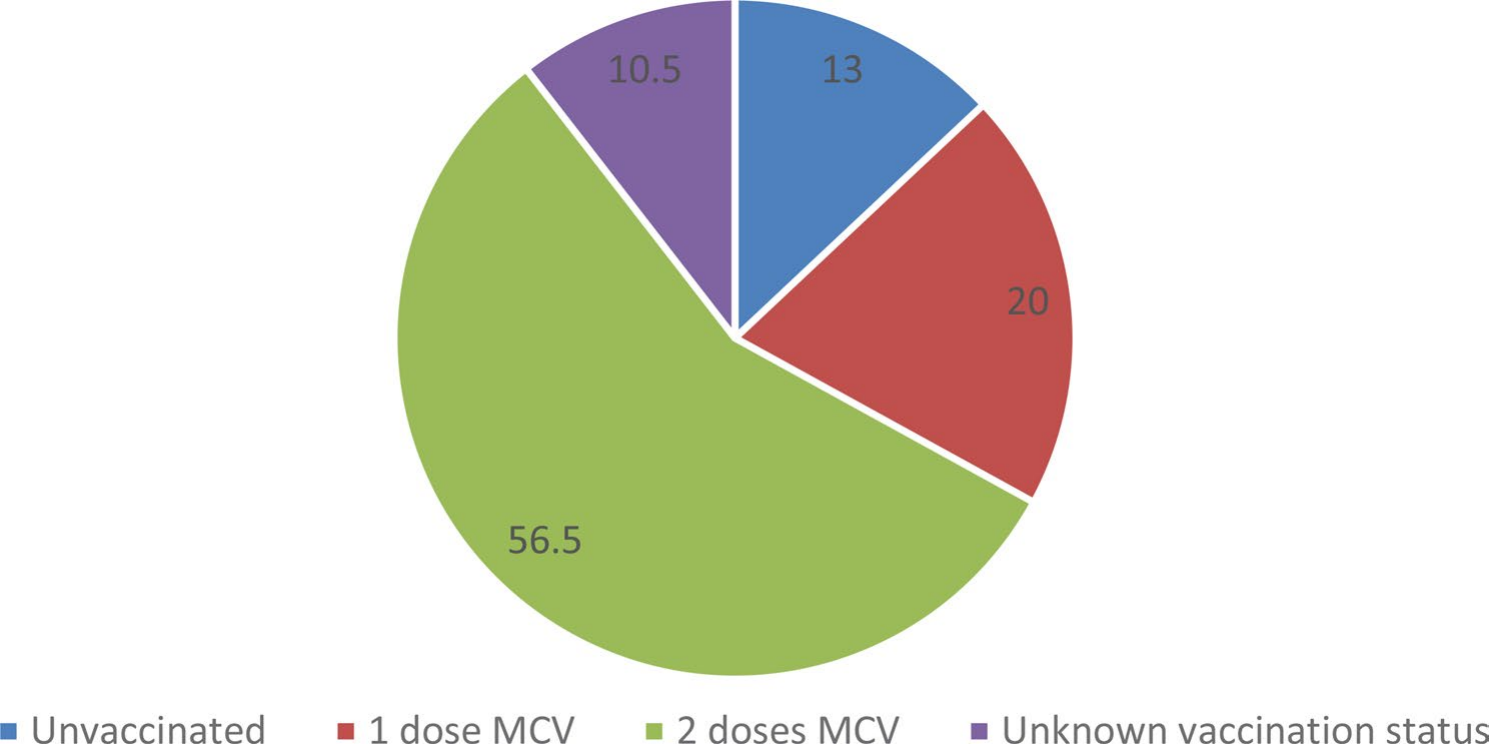
Vaccination Status of measles cases in Geze Gofa district, South Ethiopia region, 2025

Majority of the respondent (56.5%) are fully vaccinated 13% of cases are unvaccinated individuals, while 10.5% had unknown vaccination statuses. Despite high coverage rates of 96% for the first dose (MCV1) and 94% for the second dose (MCV2) vaccine from report of district health office(Fig. 3).

### Timeline of the measles outbreak

**Fig. 4:**
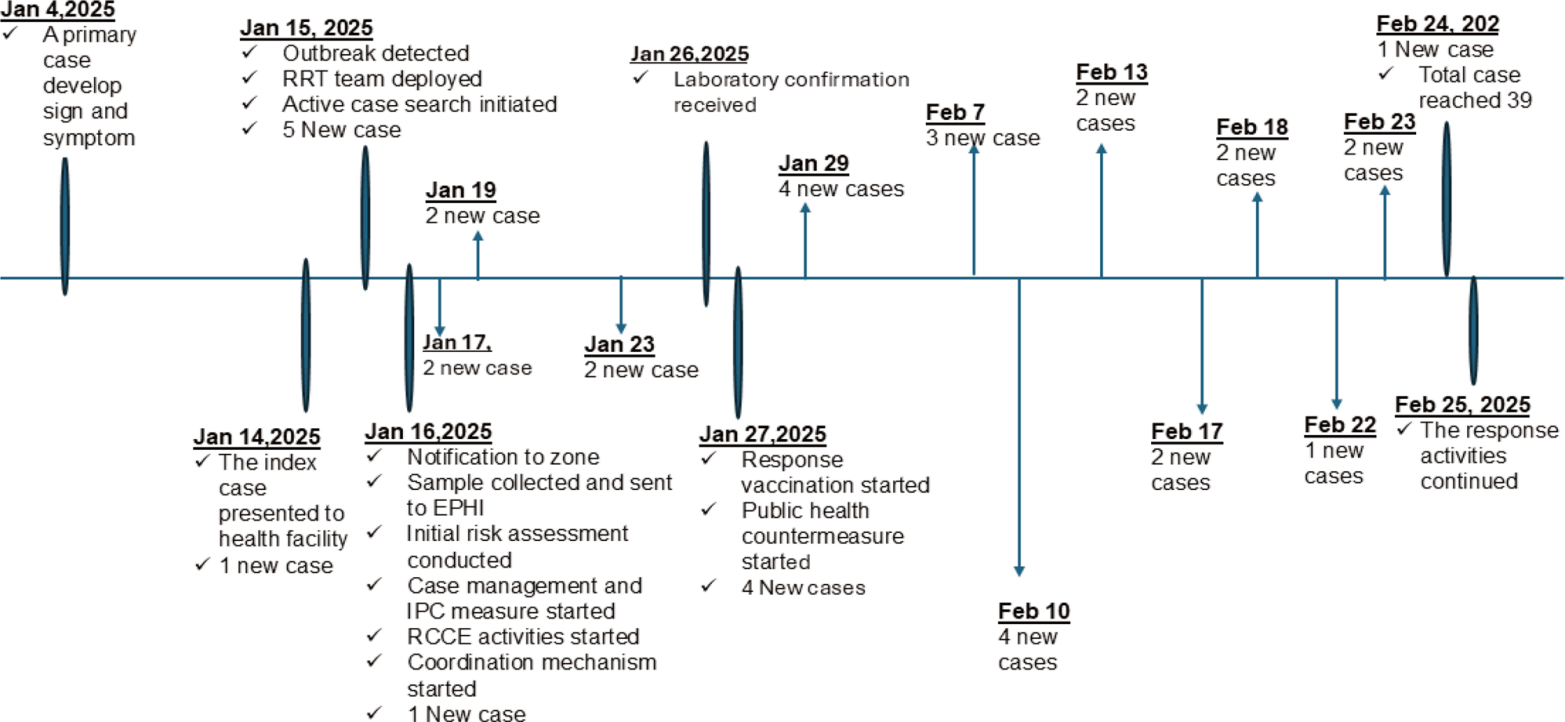
Timeline of activities done during the measle outbreak in Geze Gofa district, South Ethiopia region, February 2025

When we traced the origin of the first measles case in Geze Gofa district, we found that two family members had traveled to Dembe Gofa, a neighboring district with active measles transmission, two weeks earlier. After returning home, the mother which is suspected to be the primary case complained of a headache, a rash on her body, and redness in her eyes. Despite these issues, her symptoms were mild and resolved spontaneously. After 7 days of the mother symptom disappeared, her three years old female child (the index case) presented with four-days history of rash, fever, cough, and runny nose and went to the nearby Jawula health center. The health care provider suspected measles gave her vitamin A and notified a district health office that day and referred her due to complication to nearby hospital. After four days of treatment, she returned to her family. On the next day, three health care providers from Jawula health center gone to the household for an active case search. Then five additional measle cases were identified in that area via the active case search. The next day, the blood serum sample of those suspected cases were taken and sent to the national laboratory at Ethiopian Public Health Institute with help of district health office (Fig. 4).

The treatment was initiated, and active case searching was also continued by a catchment health worker with support from the district health office. Laboratory result of the suspected case comes on the 10th day of sent. Of the five suspected samples sent to the Ethiopian Public Health Institute, four (80%) tested positive for measles IgM antibodies. The cases of measles continued to increase up February 18, 2025, when the team gone to the area for outbreak investigation.

**Fig. 5:**
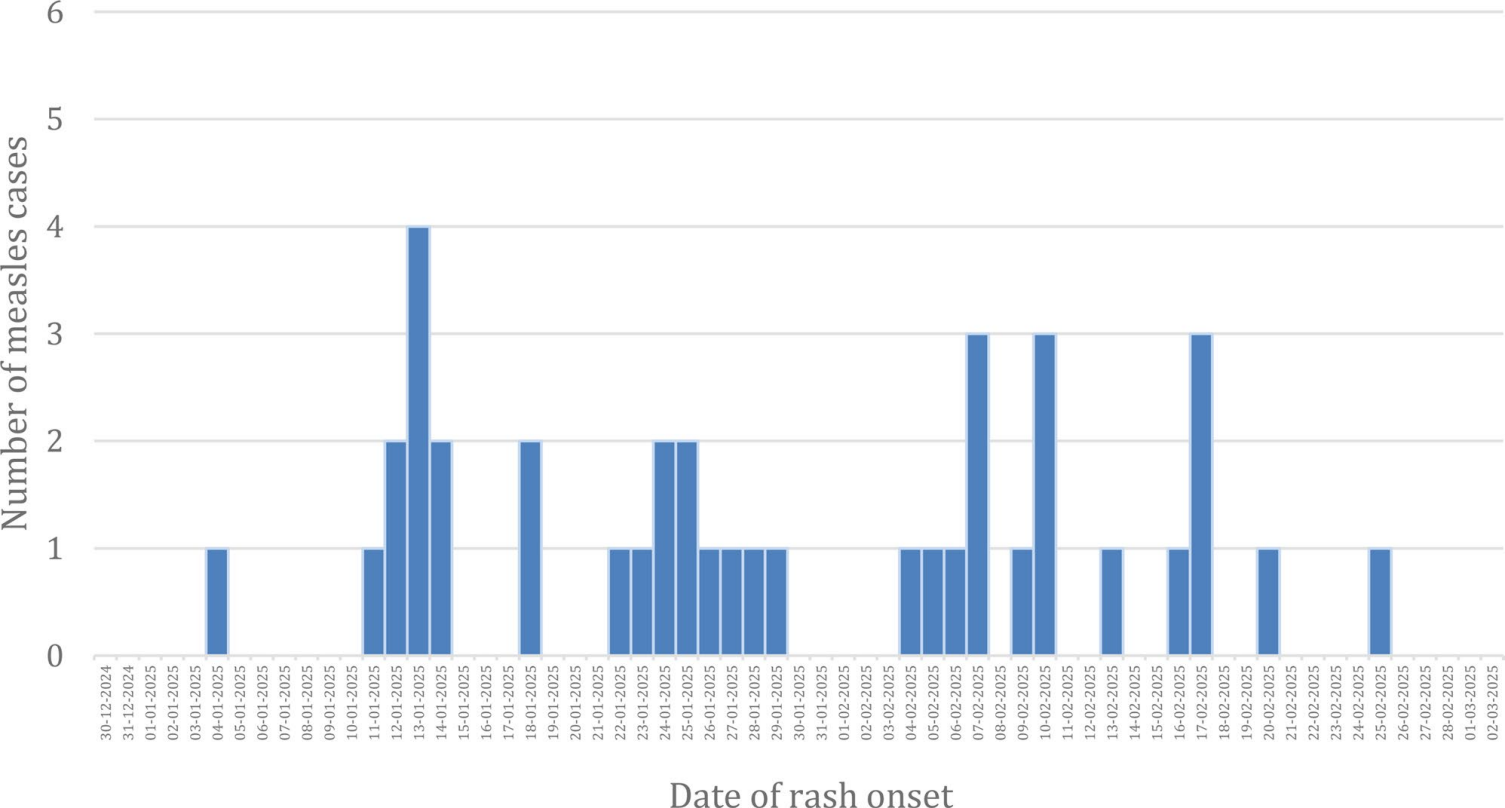
Epidemic curve of measles cases by date of rash onset, Geze Gofa district, South Ethiopia region, January–February 2025 (*n* = 39)

The epidemic curve shown represents a propagated epidemic, a type of epidemic that spreads from person to person and often showing waves of cases over time as seen in measles. The graph displays multiple peaks occurring at intervals of several days, which suggests successive waves of infection rather than a single exposure event. This pattern indicates ongoing transmission, likely through close contact within communities. The spacing between peaks aligns with the incubation period of measles (typically 10–14 days), reflecting the emergence of secondary and tertiary cases. Such a curve is commonly observed in settings where there may be delayed identification and isolation of cases, clustering of susceptible individuals, or low vaccination coverage (Fig. 5).

### 7-1-7 assessment

#### Detection

The measles outbreak was detected on January 15, 2025, when the number of suspected cases reached five, meeting the national threshold for declaring an outbreak. The date of emergence was January 4, 2025, corresponding to the onset of symptoms in the primary case, a mother. This indicates an 11-day interval between the initial emergence of the outbreak and its detection date. The health center and district health personnel suspected of measles and completed a Case Investigation Form (CIF) on the same day.

In Geze Gofa District, there are factors identified for delayed detection of the outbreak. The primary case occurred in an adult who presented with only mild symptoms that were resolved spontaneously, leading the family to overlook the possibility of measles since it is rarely anticipated in adults. Compounding this, the absence of active community-based surveillance meant that early signals were missed, allowing the disease to circulate undetected until subsequent cases emerged.

Nevertheless, valuable experiences were gained during the detection process. Health workers in the affected districts were already alert to the measles situation and consistently engaged in passive surveillance at health facilities. Following the index case which visited health facility first, they promptly initiated active case searches, which facilitated the identification of new cases. Moreover, health personnel responsible for under-five patients were aware of measles case definition and quickly recognized the symptoms, enabling them to detect the first confirmed case and strengthen active case-finding efforts.

*There was no case of measles here before and we didn’t have exposure to the cases*,* but we have case definition of the cases measles*,* but I called to the district PHEM officer and guided us a way* A health care provider at Jawula Health Center.

*Initially*,* her mother developed measles-like symptoms*,* but since the illness appeared mild*,* we did not seek care at a health facility. But when our first child became ill*,* we took her to Jawula Health Center. The health worker there treated her and referred us to Hospital due to complications. Shortly after*,* the health workers visited our home to search for other cases. They also took a sample from my daughter*,* and later informed us that she had tested positive for measles *A father of a child diagnosed with measles said

#### Notification

Following the detection of the measles outbreak, the health workers at Jawula Health Center promptly notified the cases on the same day. Interviews with the health Center team revealed that several key factors enabled this timely and effective Notification

*For case notification*,* we use mobile phones*,* which are easily accessible and always at hand. We have also been informed about the importance of immediately reporting notifiable cases*,* which helps ensure a quick response to outbreaks like measles* PHEM officer of the district.

*First*,* we informed the district PHEM officer about the measles cases when we suspect the first cases and he was following and working with us during a case search so when the thresh hold reaches*,* he immediately notifies a zonal health offices* Health care provider

#### Response

The response to the measles outbreak was initiated on the day of notification when a team from the District Health Office arrived in the area and continued until the completion of our evaluation. The response for this outbreak is done by the district teams and health worker at health centers and health posts. The Rapid Response Team of the District was deployed to investigate the situation, collect laboratory specimens, and initiate treatment while searching for other cases. They initiate public health countermeasures in affected communities like vitamin A supplementation. But a response vaccination was delayed due to waiting for outbreak confirmation which lasts 10 days to get the result of the sample taken. A response vaccination is a rapid, targeted campaign implemented during a measles outbreak to contain the spread of the virus. It focuses on immunizing all susceptible individuals, typically children aged 6 to 59 months in the affected areas, with the primary goal of breaking the chain of transmission. According to the national measles surveillance guidelines, vaccination activities are recommended after laboratory confirmation of cases or if there is epidemiological link of the case. But the response team were expecting confirmation of the case which is not in line with the national guidelines. This requirement led to delays in initiating the response vaccination. Furthermore, limited access to measles vaccines and A rugged landscape contributed to inadequate coverage, as not all eligible children within the 6 to 59 months age group were reached.

This challenge was described by the district PHEM Officer as *“The collected samples are transported and arrive in Addis Ababa two days after collection. We received the results of these five samples ten days after they were collected”*.

Similarly, “*During the recent measles outbreak response*,* vaccination was delayed because teams were awaiting laboratory confirmation before initiating immunization for suspected cases. However*,* this approach was inconsistent with national guidelines*,* which allow vaccination to begin based on epidemiological linkage alone. We have since recognized that laboratory confirmation is not mandatory when there is clear evidence of transmission or exposure and there is scarcity of a measles vaccine to caver entire less than five populations that is why the coverage is 52 presents’’* PHEM officer at Jawula health center.

*Regarding to training on public health emergency management only the district PHEM officer has front line field epidemiology training and the health worker at facility level has no training related to this topic* District health Office manager.

*Lack of enough health workers at health post for example one of our four health post didn’t have health extension worker for a long time due to educational opportunity at this time population on the area lack enough service that they should have to get* PHCU of the Jawula Health center.

*As you can observe*,* the landscape of our district is challenging and difficult to cover with the available workforce. This geographical barrier significantly hinders our ability to reach all children for vaccination*,* as the logistical constraints further complicate the delivery of vaccines to remote areas* health care provider at Bedro health center.

The summary of measle outbreak response, detection, and response is presented in Table 2.

**Table 2:** Timelines of measles outbreak at Geze Gofa district, South Ethiopia region using the 7–1–7 matrix, February 2025

Steps of investigation	Time range of the steps	Timeliness in days	Target in days	Met target?
1. Detection	Difference between dates of emergence and detection	11	7	No
2. Notification	Difference between dates of detection and notification	1	1	Yes
3. Response	Difference between dates of notification and initiation of the last early response action	11	7	No

### Result from observation

On observation significant gap was noted in vaccination card. Most of the children with suspected measles did not possess vaccination cards, and among those who did, many cards were either incomplete or improperly filled out. This indicates a potential issue in both vaccination coverage and record-keeping, which may hinder effective outbreak response and monitoring efforts.

## Discussion

The objective of this study was to investigate measles outbreak in Geze Gofa district and evaluate the response efforts, identifying both bottlenecks and enabling factors. The outbreak management in Geze Gofa District met the 7-1-7 targets for notification (1 day) however, the detection [12] and early response initiation was delayed (11 days), exceeding the WHO recommended timeframe.

A total of 39 suspected and confirmed cases were reported over seven weeks with no fatalities. The propagated epidemic curve and geographic clustering, particularly in Jawula Wuga kebele, indicate sustained person-to-person transmission within household and communities. Similar outbreak patterns have been documented in Ethiopia and other sub-Saharan African settings, where under-immunized populations and community clustering amplify measles spread communities [21, 22]. The predominance of cases among children aged 1–14 years, especially school-age children, aligns with known measles epidemiology, reflecting the role of partially immunized children as primary drivers of transmission [21].

Our findings indicate that the index case was likely exposed to measles by her mother, who developed mild, self-limited symptoms after contracting the disease during a visit to an outbreak-affected district. This highlights the role of human mobility in the spread of infectious diseases, especially in regions with porous district boundaries and strong social ties that prompt frequent travel. These factors increase the likelihood of disease transmission and pose challenges for containment efforts [23–25]. A systematic review that synthesized findings of nearly 200 studies across various pathogens found a strong association between human movement (local and long-distance) and spread of infectious diseases, especially respiratory illnesses [24]. A recent study on comparing and integrating human mobility data sources for measles transmission modeling in Zambia demonstrated that higher mobility leads to introduction of measles in many more districts, underlining how travel can drive outbreaks in previously unaffected or controlled areas [25]. In communities with strong social ties, people interact closely and frequently. These connections often lead to more movement between neighboring areas, which changes how populations mix. As a result, there is a greater chance that susceptible individuals will meet those who are infectious across district or geographic boundaries.

The identification of the primary case as an adult with mild, self-resolving symptoms highlights a common oversight of measles in adults. Given that measles is often perceived as a childhood disease, families may not readily associate mild symptoms with potential measles infection [26–28]. This misconception can lead to delayed health facility seeking as seen in this case. Delayed diagnosis occurred because adult measles is uncommon and symptoms were non-specific, which is consistent with the idea that adults and providers may not immediately associate mild presentation with measles infection [28].

The lack of active community-based surveillance mechanisms significantly hindered early detection of the disease. Surveillance systems that engage communities in health monitoring are crucial for identifying outbreaks before they escalate. In this instance, the absence of such systems allowed measles to circulate unnoticed, ultimately resulting in additional cases. Community-based measles mortality surveillance in Congo support this [29]. The situation emphasizes the need for robust surveillance frameworks that include community participation, enabling timely identification and response to potential disease outbreaks before widespread transmission occurs.

Despite the delay, the response to the index case demonstrated the resilience and adaptability of health workers in the region. The initial identification of the case through routine passive surveillance, followed by a prompt shift to active case finding, reflects a functioning preparedness and response mechanism at the local level. Such responsiveness underscores the critical role of trained and alert frontline health workers in detecting early signals of outbreaks, initiating timely public health actions, and limiting onward transmission. Similar observations have been documented in outbreak investigations, where early recognition by health workers and rapid escalation from passive to active surveillance were pivotal in controlling measles and other epidemic-prone diseases, particularly in resource-constrained settings [30–32]. Additionally, the rapid recognition of measles symptoms by healthcare personnel working with under-five patients highlights the effectiveness of targeted training. Ensuring that frontline workers are familiar with case definitions improves early detection and containment of outbreaks.

Despite the delays in timely detection of the case, the early notification reflects surveillance awareness at the primary care unit (health center) level. This can be attributed to prior sensitization about measles due to nearby outbreaks, familiarity with case definitions, and effective use of communication tools such as mobile phones. Health workers quickly identified symptoms, initiated a case investigation, and promptly notified the district PHEM officer, triggering an initial response. These strengths illustrate how even resource-limited settings can demonstrate resilience when surveillance systems are functional and health workers are empowered [33, 34]. A case study from Burkina Faso indicated that community-based health workers (CHWs) can successfully perform epidemic disease surveillance, including early detection and reporting. Moreover, the study demonstrated that empowering CHWs contributes to a functioning surveillance system even in low-resource settings [34]. A quasi-experimental study in Tanzania demonstrated that training frontline workers and providing them e-based reporting tools improved disease reporting in a pastoral, resource-poor context [33]. Similarly, a pilot study in rural Kenya also demonstrates that mobile-phone–based reporting by health workers or community volunteers can meaningfully contribute to early disease detection in resource-limited areas [29]. Enhanced use of mobile technology for rapid case reporting and real-time communication among health officials can improve the timeliness and efficiency of outbreak responses. In addition, surveillance data sharing across district borders should be strengthened to anticipate and mitigate cross district transmission.

Although the outbreak was notified in a timely manner in line with the 7-1-7 targets, the district failed to meet the seven-day benchmark of the 7-1-7 framework for initiating a full response. Significant bottlenecks were the delay in laboratory confirmation and delayed response vaccination due to knowledge gap on how and when to give a response vaccination. While laboratory confirmation is vital for accurate diagnosis, this limited the district’s ability to rapidly respond, particularly in the context of highly transmissible diseases like measles. This pattern of delayed response vaccination observed in our setting is consistent with findings from other outbreak investigations in Ethiopia. For example, an unmatched case–control study conducted in Tocha district, southwestern Ethiopia, reported that outbreak-response measures were initiated only after the index cases had already died, illustrating how delays in response vaccination can severely undermine outbreak control [21]. Moreover, the delay in implementing an outbreak-response vaccination campaign substantially increases the probability of a large outbreak. Even delay of a few weeks markedly reduce the effectiveness of the vaccination campaigns; whereas early response approximately within one week minimizes outbreak size [35]. Delayed response vaccination not only hindered containment efforts but also resulted in suboptimal coverage, reaching only 52% of the target population. Together, these findings reinforce that early detection, timely laboratory support, and rapid initiation of response vaccination are critical for effective measles outbreak control and for meeting the performance benchmarks of the 7-1-7 framework. In addition, laboratory testing capacity should be decentralized, and turnaround times significantly reduced. Strengthening regional laboratories would help minimize delays associated with sample transportation and result reporting.

In addition to delayed lab feedback, the investigation revealed systemic issues that impeded effective response. One health post lacked a health extension worker due to staff attrition and slow replacement. Moreover, only one district-level staff member had frontline field epidemiology training in public health emergency management, severely limiting capacity for coordinated outbreak control. A multicounty analysis on human resource challenges in health systems based on evidence from 10 African countries showed that many health centers and health posts in sub-Saharan Africa operate with one or no clinical health workers after adjusting for absenteeism [36]. This finding indicated widespread understaffing at the primary care level that undermined consistent service delivery and outbreak response capacity [36]. The broader sub-Saharan Africa epidemiology capacity literature highlighted that diagnostic capacity, coordination, logistic support, and trained workforce are recurrent constraints impairing timely outbreak response [22]. Hence, human resource capacity must also be addressed by strengthening capacity building activities for frontline surveillance officers, health center staff and health extension workers to improve outbreak detection and response.

Geographical barriers added further complexity. The rugged terrain of the district made it difficult for health workers to reach remote areas, especially during mass vaccination efforts. Combined with limited vaccine availability, this led to reduced coverage and increased risk of further transmission. This finding was supported by a study in Africa that demonstrated “remoteness” as approximated by travel time to urban centers or health-service points is strongly associated with lower measles vaccine coverage across 26 African countries [37]. The national-level spatial analysis of immunization coverage in Ethiopia found that travel time to nearest city was a negative predictor of effective immunization coverage. Those children in remote areas had lower effective coverage. Moreover, geographical disparities including distance and infrastructure constraints were among the main challenges to equitable vaccine delivery in the resource limited areas [38]. Therefore, improving vaccine logistics is essential particularly at remote district level. Stockpiling measles vaccines at zonal and district levels would ensure rapid deployment during outbreaks. In addition, cold chain and distribution systems should be strengthened to guarantee that vaccines can reach even the most remote communities without compromise.

Health facilities-initiated treatment promptly, including vitamin A supplementation, which has been associated with reductions in measles-related morbidity and mortality in some settings [39]. Active case search efforts at health centers and through household visits helped uncover additional cases early. Community cooperation and trust in the health system also played an important role in facilitating rapid reporting and case management.

This study has some limitations that should be considered when interpreting the findings. First, it was based on a single outbreak in Geze Gofa District and used a purposive sampling approach, which may not be fully represent the other regions of Ethiopia that may have different health system capacities, geographic challenges, or population dynamics. Second, the study relied on interviews and self-reported information from caregivers and health workers, which could introduce recall bias and affect the accuracy of details on vaccination status and outbreak timelines. To mitigate this limitations, broader multi-districts future research should be conducted using probabilistic sampling methods and by triangulating self-reported data with vaccination registries and laboratory records to enhance the validity and reliability data. Despite these limitations, the study provides valuable insights into outbreak response challenges and highlights practical opportunities for strengthening surveillance and vaccination strategies in similar low-resource settings.

## Conclusion

The measles outbreak in Geze Gofa district highlighted that the district’s public health response system performed commendably in case notification, while delays in detection and full implementation of response actions revealed systemic bottlenecks in outbreak management. Challenges such as delayed laboratory confirmation, insufficient vaccine availability and difficult geographic access hampered the timely control of the outbreak.

## Supplementary Information

Below is the link to the electronic supplementary material.

Supplementary Material 1

## Data Availability

Data upon which the result is based will be available from the corresponding author upon request.

## Abbreviations

CDC: Africa center for disease control and prevention
CIF: Case investigation form
EPHI: Ethiopian public health institute
EPI: Expanded program on immunization
IPC: Infection prevention and control
MCV1: Measles containing vaccine, first dose
MCV2: Measles containing vaccine, second dose
MoH: Ministry of health
PHCU: Primary health care unit
PHEM: Public health emergency management
RCCE: Risk communication and community engagement
RRT: Rapid response team
SIAs: Supplementary immunization activities
SSPE: Subacute sclerosing panencephalitis
WHO: World Health Organization
