# Supplementary material for: Evaluation of measles outbreak response in Geze Gofa district, South Ethiopia region, using the 7–1–7 timeliness metrics
Source: BMC Infect Dis. 2026 Apr 16;26:1053. doi: 10.1186/s12879-026-13299-2 (PMC13224625; doi:10.1186/s12879-026-13299-2)
Supplement: Supplementary file 1 — Supplementary Material 1 [file 12879_2026_13299_MOESM1_ESM.docx]

**Measles Outbreak Timeline and Early Response Documentation Tool of Geze gofa district, February 2025**

1. **Step 1. Record dates for milestones**

| Milestones | Date  DD/MM/YYYY | | Narrative Briefly describe key observations in this interval and how the date was determined. | |
| --- | --- | --- | --- | --- |
| Date of emergence | | | | |
| Question 2.1: What was the symptom onset date for the first suspected case?  *Measles suspected case definition:* *Any person with fever and maculopapular (non- vesicular) generalized rash and cough, coryza or conjunctivitis (red eyes) or any person in whom a clinician suspects measles.* | |  | |  |
|  | |  | |  |
| Question 2.2: For the current/ongoing outbreak when was the action threshold crossed? *Action threshold: 5 suspected or 3 confirmed cases (Take whichever happened first and indicate which one is being referred to in the narrative box)*   - *If interviewee is unable to recall the date, suggest for them look up the dates from the line list data or weekly aggregate reporting excel sheet.* | |  | |  |
| Date of detection | | | | |
| Question 2.3: On what date did you recognize that the action threshold has been crossed? | | DD/MM/YYYY | |  |
| Date of Notification | | | | |
| Date of notification to woreda PHEM  Question 2.4: Were you notified of the first suspected case from the health facility? If yes, on what date? | |  | |  |
| Date of notification to zonal PHEM (if applicable)  Question 2.4: Did you notify the zonal PHEM? If yes, on what date did you notify the zonal PHEM? | |  | |  |
| Date of notification to regional PHEM  Question 2.4: Did you notify the regional PHEM? If yes, on what date did you notify the regional PHEM? If no, do you know when the regional PHEM was notified by the zone? | |  | |  |
| Date of notification to EPHI  Question 2.4: Did you notify EPHI PHEM directly? If no, skip. If yes, on what date did you notify the regional PHEM? | |  | |  |
| Date of Early Response Actions | | | | |
| Question 2.5: When did you initiate investigation or deploy investigation/response team? | |  | |  |
| Question 2.6: When did you conduct epidemiologic analysis of burden, severity and risk factors, and perform initial risk assessment  (*Describe in the narrative activities conducted)* | |  | |  |
| Question 2.7: When was laboratory confirmation obtained? | |  | |  |
| Question 2.8: When did you initiate appropriate case management and infection prevention and control (IPC) measures in health facilities *(Activities may include case management SOP distribution/training, isolation room set up etc.)* | |  | |  |
| Question 2.9: When did you initiate appropriate public health countermeasures in affected communities *(E.g., Initiated procurement and distribution of commodities in the community to prevent outbreak spread (e.g., vaccines, antibiotics, vitamin A)* | |  | |  |
| Question 2.10: When did you initiate appropriate risk communication and community engagement activities | |  | |  |
| Question 2.11: When did you establish a coordination mechanism? *(E.g., activation of multisectoral taskforce)* | |  | |  |
| Summary of Early Response Initiation and Completion (to be filled by interviewer) | | | | |
| Date of early response initiation Date on which the first of the seven early response actions occurred (see above) | |  | |  |
| Date of early response completion Date on which all applicable early response actions were completed (see above) | |  | |  |

**Step 2. Calculate timeliness in 7-1-7 intervals**

| Interval | Calculation  In days | Timeliness  In days | Target  In days | Met target?  Yes/No |
| --- | --- | --- | --- | --- |
| Detection | Difference between dates of emergence and detection |  | 7 |  |
| Notification | Difference between dates of detection and notification |  | 1 |  |
| Response | Difference between dates of  notification and  completion of the last early response action |  | 7 |  |

**Step 3. Identify bottlenecks and enablers**

| Interval | Bottlenecks  Factors that prevented timely action.  Identify max 3, if applicable. Propose remedial actions in Step 4. | Enablers  Factors that enabled timely action.  Identify max 3, if applicable. Document for advocacy and to demonstrate impact. |
| --- | --- | --- |
| Detection |  |  |
| Notification |  |  |
| Response |  |  |

1. **Quantitative questionnaire for cases of Measles**

**Case Identification**

1. Respondent ID…...
2. Date of interview…….
3. District…………….Kebele……………Got…………….
4. Age in months if under 5…………….

In Year if greater than or equal to 5 years……….

1. Sex: Male …... Female……...
2. Date of symptom onset……...
3. Symptoms: Fever…. Rash……... Cough…...Conjunctivitis…… Coryza…….

**Vaccination History**

1. Have you received measles vaccine? Yes… No……Unknown…….
2. Number of doses received………………

**Exposure History**

1. Any travel in the past 3 weeks? Yes …. No………

If yes, where?

1. Contact with known measles case. Yes…. No…….
2. Attended school, market, religious gathering, or other crowded places recently? Yes ……. No…….

If yes, specify………….

**Laboratory Investigation**

1. Specimen collected? Yes……. No……….
2. Lab result: Positive…. Negative…Pending….
